# A rare case report: retroperitoneal dedifferentiated liposarcoma associated with paraneoplastic pemphigus

**DOI:** 10.3389/fsurg.2025.1690860

**Published:** 2025-10-17

**Authors:** Fan Yang, Yangju Chen, Jiaqing Liu, Tingyu Gu, Queping Song, Xiao Zhou, Fuan Xie, Han Gao, Xueling Wang, Qin Zhang, Xiaogang Xia, Qing Wang, Wengang Li

**Affiliations:** 1 Cancer Research Center, School of Medicine, Xiamen University, Xiamen, China; 2 Department of Hepatobiliary Surgery, Xiang’an Hospital of Xiamen University, Xiamen, China; 3 Department of Neurology, Zhongshan Hospital (Xiamen), Fudan University, Xiamen, China; 4 Department of Radiation Oncology, the First Affiliated Hospital of Xiamen University, School of Medicine, Xiamen University, Xiamen, Fujian, China; 5 Xiamen Cancer Quality Control Center, Xiamen, Fujian, China; 6 Xiamen Cancer Center, Xiamen, Fujian, China; 7 Department of Health Medical Center, Xiang'an Hospital of Xiamen University, School of Medicine, Xiamen University, Xiamen, China; 8 Department of Otolaryngology-Head and Neck Surgery, Xiang’an Hospital of Xiamen University, Xiamen, China; 9 Department of Gastroenterology, Xiang'an Hospital, Xiamen University, Xiamen, Fujian, China

**Keywords:** dedifferentiated liposarcoma, retroperitoneal neoplasm, paraneoplastic pemphigus, autoimmune blistering disease, case report

## Abstract

**Background:**

Dedifferentiated liposarcoma (DDLPS) is a rare and aggressive malignant tumor, particularly when occurring in the retroperitoneum. Paraneoplastic pemphigus (PNP) is an uncommon autoimmune mucocutaneous disorder often associated with neoplasia. The coexistence of retroperitoneal DDLPS and PNP is exceptionally uncommon, with only sporadic cases reported worldwide. We present a case of retroperitoneal DDLPS with concurrent PNP in a young male, emphasizing diagnostic challenges and management strategies.

**Case presentation:**

A 24-year-old male presented with a one-year history of refractory oral ulcerations and a four-month history of a retroperitoneal mass. Initial workup at an outside hospital led to a diagnosis of pemphigus vulgaris, and corticosteroid therapy was initiated without significant improvement. CT demonstrated a large retroperitoneal mass encasing the right iliac vessels and inferior vena cava. Preoperative embolization was performed, followed by complete surgical resection, radiofrequency ablation of residual tumor, and right ureteral stent placement. Histopathology confirmed DDLPS with inflammatory infiltration. Postoperatively, this patient's mucocutaneous lesions improved with continued corticosteroids and topical care.

**Conclusion:**

This rare presentation underscores the importance of recognizing paraneoplastic autoimmune syndromes as potential indicators of underlying malignancy. Complete tumor resection remains the cornerstone of management, and multidisciplinary care is essential to optimize both oncologic and autoimmune outcomes.

## 1 Introduction

Dedifferentiated liposarcoma (DDLPS) accounts for approximately 10%–15% of retroperitoneal sarcomas and is characterized by the abrupt transition from well-differentiated liposarcoma to a non-lipogenic sarcoma of variable histologic grade (1). Owing to its deep location and nonspecific early symptoms, most patients present at an advanced stage (2). Standard treatment involves complete surgical excision; however, local recurrence is common, and response to radiotherapy and chemotherapy is limited (2). Paraneoplastic pemphigus (PNP) is a rare autoimmune mucocutaneous disease characterized by autoantibodies targeting desmosomal proteins (3, 4). While commonly associated with lymphoproliferative malignancies, PNP may also occur with solid tumors (5, 6). The coexistence of retroperitoneal DDLPS and PNP is exceptionally rare, with only isolated cases reported (7). We report a case of retroperitoneal DDLPS with PNP in a young male, focusing on the clinical features, diagnostic approach, surgical management, and review of current literature.

## 2 Case description

A 24-year-old male presented to our hospital in December 2024 with a history of one-year history of persistent, painful oral ulcers and a four-month history of a retroperitoneal mass. The oral lesions initially responded to symptomatic treatment but recurred with progressive skin desquamation of the hands and feet, erythema, and pruritus. Four months prior, abdominal CT performed at an outside hospital demonstrated a retroperitoneal mass; no further treatment was undertaken at that time. The patient was diagnosed with pemphigus vulgaris and started on methylprednisolone. He had no significant past medical history, allergies, or family history of malignancy.

**Figure 1:**
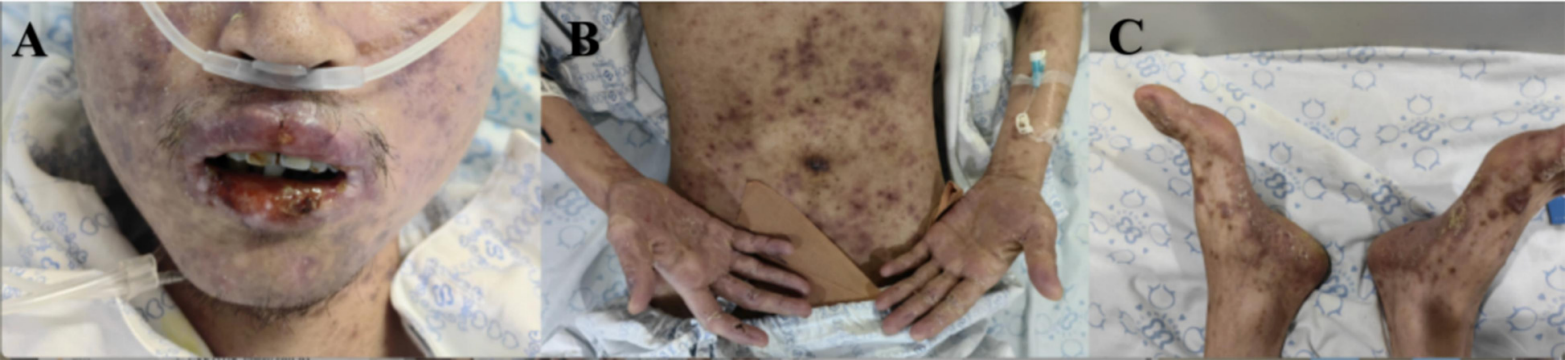
Erosions of the oral mucosa **(A)**, dark red maculopapular rash on the trunk **(B, C)** and desquamation on multiple areas of the hands and feet **(B, C)**.

On examination, his vital signs were stable. Dermatological evaluation demonstrated multiple areas of desquamation on the hands and feet, dark red maculopapular rashes on the trunk, and extensive erosions of the oral mucosa (Figure 1).

**Figure 2:**
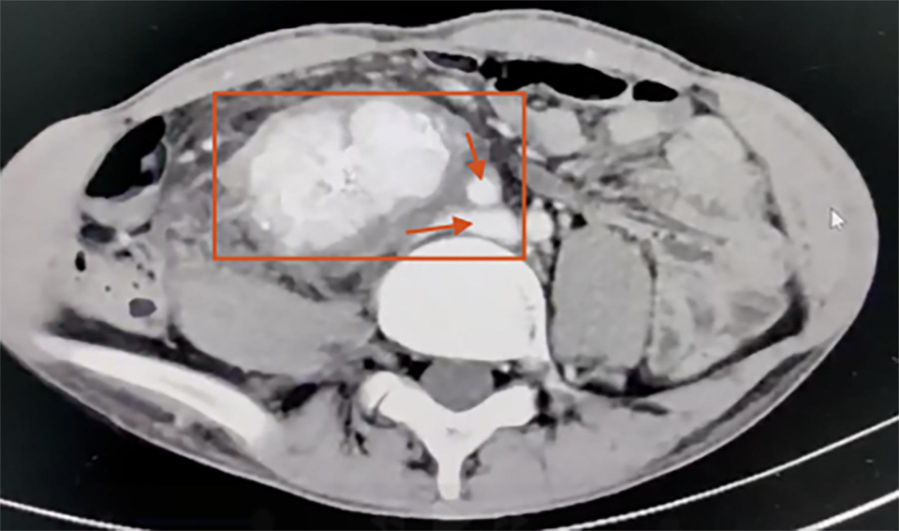
Contrast-enhanced abdominal CT scan demonstrating a malignant retroperitoneal mass (17 × 10 × 4 cm) encasing the right iliac vessels and inferior vena cava (the red box highlights a retroperitoneal malignant mass. The superior arrow indicates encasement of the right iliac vessels, while the inferior arrow indicates encasement of the inferior vena cava).

Laboratory tests revealed leukocytosis with a white blood cell count of 12.13 × 10^9^/L (reference range: 3.5–9.5 × 10^9^/L, ↑), neutrophilia with neutrophils accounting for 92.4% (reference range: 40%–75%, ↑), hemoglobin 153 g/L (reference range: 130–175 g/L, normal), and platelet count 368 × 10^9^/L (reference range: 125–350 × 10^9^/L, ↑). Serum biochemistry demonstrated decreased prealbumin at 160 mg/L (reference range: 200–400 mg/L, ↓), decreased albumin at 32.9 g/L (reference range: 40–55 g/L, ↓), and hypokalemia with potassium at 3.09 mmol/L (reference range: 3.5–5.3 mmol/L, ↓). Tumor marker analysis revealed an elevated carcinoembryonic antigen (CEA) level of 7.170 ng/ml (reference range: 0–5 ng/ml, ↑). Coagulation studies showed a mildly prolonged prothrombin time–international normalized ratio (PT-INR) of 1.27 (reference range: 0.8–1.2, ↑) and elevated plasma fibrinogen at 5.70 g/L (reference range: 2–4 g/L, ↑). Contrast-enhanced CT demonstrated a 17 × 10 × 4 cm retroperitoneal mass encasing the right iliac vessels and inferior vena cava (Figure 2) consistent with liposarcoma.

**Figure 3:**
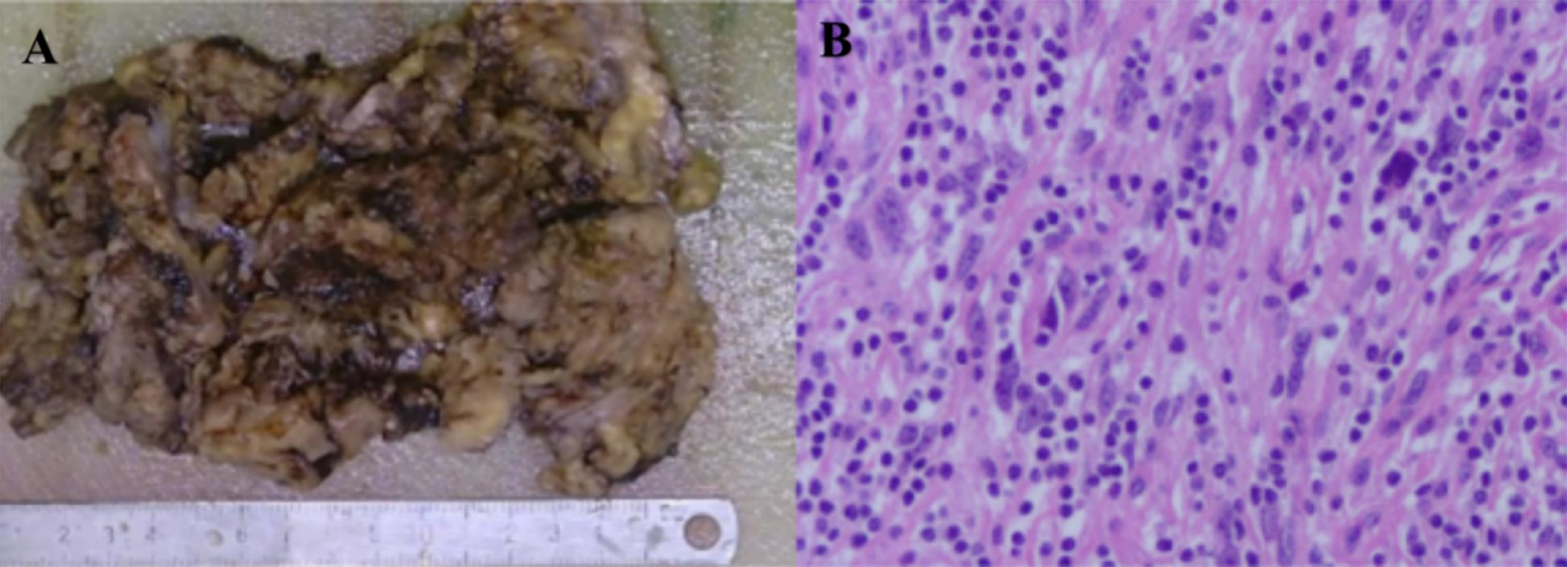
Histopathological examination demonstrating spindle-shaped tumor cells arranged in fascicles with nuclear atypia, lymphocytic infiltration in the stroma, and thick-walled vessels with calcification **(A, B)**.

On December 24, 2024, the patient underwent right internal iliac artery embolization to reduce tumor vascularity (1 h 10 min, 10 ml blood loss, no transfusion). The following day, open resection of the retroperitoneal mass was performed, combined with radiofrequency ablation of residual lesions (Monopolar ablation was delivered at 50–60 W with a target temperature of 60–70 °C, 1.5–2 min per site. Completeness of ablation was verified by visual inspection, palpation, thermal monitoring, immediate postoperative ultrasound, and pathological assessment, ensuring effective elimination of residual disease while protecting surrounding structures.) and right ureteral stent placement (6 h 10 min, 500 ml blood loss), during which 2 units of red blood cells and 600 ml of plasma were transfused. The resected specimen measured 10 × 8 cm, partially involving the psoas major muscle and the implanted ureteral stent. The tumor had firm consistency, poorly defined margins, and invaded the right iliac vessels, inferior vena cava, abdominal aorta, psoas major muscle, and mesenteric root. Complete resection was achieved with R0 margins confirmed, and histopathology showed spindle cell proliferation arranged in intersecting fascicles, nuclear atypia, and thick-walled vessels with calcification, consistent with dedifferentiated liposarcoma (DDLPS) (Figure 3).

Postoperatively, the patient experienced Clavien–Dindo Grade II complications, including hypoxemia requiring tracheal intubation and ICU ventilator support, transient coagulopathy, and hypoproteinemia/electrolyte imbalance, all corrected via supplementation. The patient remained in the ICU from December 25, 2024, to January 6, 2025, underwent tracheotomy on January 7, 2025, and was subsequently transferred back to the general ward.

Concurrently, paraneoplastic pemphigus was managed with oral methylprednisolone stabilized at 4 mg/day, without a physician-guided taper during hospitalization. No adjunctive immunosuppressive therapies, such as IVIG or rituximab, were administered, considering the moderate severity of symptoms and perioperative infection risks. Mucocutaneous lesions and pressure ulcers were treated with a multidisciplinary approach, including topical corticosteroids, antimicrobial rinses, epidermal growth factor spray, hydrocolloid dressings, and nutritional support via nasojejunal feeding. Clinical improvement closely followed tumor resection: oral mucosal erosions began healing within the first postoperative week, trunk and limb erythema and desquamation improved in the second week, and complete resolution of mucocutaneous lesions and pressure ulcers was achieved by three weeks, supporting the link between tumor removal and autoimmune symptom resolution.

## 3 Discussion

This report presents an exceptionally rare case of a young male with concurrent retroperitoneal dedifferentiated liposarcoma (DDLPS) and paraneoplastic pemphigus (PNP). DDLPS is an aggressive, high-grade soft tissue sarcoma that often remains asymptomatic in its early stages due to its deep retroperitoneal location, typically being diagnosed only when it has reached a considerable size and involves major vessels or adjacent organs. PNP, in contrast, is a rare autoimmune blistering disorder more commonly linked to lymphoproliferative malignancies, and its co-occurrence with soft tissue sarcomas—especially retroperitoneal DDLPS—is extraordinarily uncommon. In this case, the patient initially presented with persistent oral ulcers and progressive cutaneous lesions, which prompted further imaging that revealed a large retroperitoneal mass encasing the iliac vessels and inferior vena cava. Complete surgical resection not only achieved oncologic control but also resulted in a marked improvement in PNP symptoms. This case underscores the diagnostic challenges posed by such rare tumor–autoimmune disease associations and highlights the importance of integrated treatment strategies to address both malignancy and paraneoplastic manifestations (8). In addition, it is noteworthy that carcinoembryonic antigen (CEA) was the only tumor marker tested and was found to be elevated (1). Although CEA is not a specific biomarker for soft tissue sarcomas, its elevation in this patient may reflect tumor burden or systemic inflammation, consistent with reports of nonspecific CEA elevation in certain mesenchymal malignancies. Therefore, its inclusion in this case provides additional clinical context.

The clinical implications of PNP extend beyond cutaneous and mucosal lesions, as it may serve as an early warning sign of an underlying malignancy. In this patient, PNP co-occurred with retroperitoneal DDLPS, and radical tumor resection resulted in marked improvement of autoimmune symptoms. Multidisciplinary collaboration—including surgical oncology, dermatology, and medical oncology—was essential to achieve optimal outcomes, balancing tumor control with management of PNP.

The pathogenesis of PNP in this patient is presumed to be tumor-driven, as substantial improvement in mucocutaneous symptoms was observed following complete tumor resection. This temporal relationship supports the role of the retroperitoneal DDLPS as the trigger for PNP in this case.

## 4 Conclusion

This case illustrates the diagnostic complexity and therapeutic challenges posed by the exceptionally rare coexistence of retroperitoneal dedifferentiated liposarcoma and paraneoplastic pemphigus in a young adult. The patient's presentation with refractory mucocutaneous lesions served as a critical clinical clue to the underlying malignancy. Complete surgical resection not only achieved oncologic control but also led to marked improvement in autoimmune manifestations, highlighting the pivotal role of tumor removal in the management of PNP. This case underscores the necessity of maintaining a high index of suspicion for paraneoplastic syndromes in patients with unexplained dermatologic or mucosal disease (9, 10), particularly when resistant to conventional therapy. Optimal management requires a multidisciplinary approach that integrates surgical, oncologic, and immunologic expertise (11) Further research is needed to elucidate the immunopathogenic links between soft tissue sarcomas and PNP (12), as well as to develop targeted strategies that address both tumor progression and autoimmune activity (13, 14).

### 4.1 Limitation statement

A major limitation of this case is the lack of long-term follow-up information. The patient's family declined subsequent clinical visits and imaging surveillance, preventing further assessment of oncologic recurrence or the remission/relapse status of paraneoplastic pemphigus.

## Data Availability

The original contributions presented in the study are included in the article/Supplementary Material, further inquiries can be directed to the corresponding authors.
